# Excellent Strength–Impact Toughness Combination of Heterostructured Metastable Fe-Rich Medium-Entropy Alloy

**DOI:** 10.3390/ma18030476

**Published:** 2025-01-21

**Authors:** Dmitrii Panov, Ruslan Chernichenko, Stanislav Naumov, Egor Kudryavtsev, Alexey Pertcev, Nikita Stepanov, Sergey Zherebtsov, Gennady Salishchev

**Affiliations:** 1 Laboratory of Bulk Nanostructured Materials, Belgorod State University, 308015 Belgorod, Russia; chernichenko@bsuedu.ru (R.C.); naumov_s@bsuedu.ru (S.N.); kudryavtsev@bsuedu.ru (E.K.); stepanov@bsuedu.ru (N.S.); zherebtsov@bsuedu.ru (S.Z.); salishchev_g@bsuedu.ru (G.S.); 2 Department Chief Metallurgist, Perm Scientific-Research Technological Institute, 614990 Perm, Russia; ogmet@pniti.ru; 3 World-Class Research Center “Advanced Digital Technologies”, State Marine Technical University, 198095 Saint-Petersburg, Russia

**Keywords:** medium-entropy alloy, heterogeneous structure, texture, cold rotary swaging, post-deformation annealing, maximum stress, Charpy impact toughness

## Abstract

The effect of a heterogeneous structure obtained via cold rotary swaging (CRS) and post-deformation annealing (PDA) on the dynamic mechanical properties of a non-equiatomic 49.5Fe-30Mn-10Co-10Cr-0.5C (at.%) medium-entropy alloy at room and cryogenic temperatures was studied. CRS to a reduction of 92% and subsequent PDA at 500–600 °C developed a heterogeneous structure consisting of a twinned γ-matrix and dislocation-free γ-grains in the rod core and an ultrafine-grained microstructure of γ-phase at the rod edge. Therefore, the maximum stress (σ_m_) value increased. Charpy V-notch impact toughness (KCV) decreased after CRS to a reduction of 18% and stabilized after further straining. However, the contribution of the crack initiation energy consumption (KCV_i_) increased, while the crack propagation energy consumption (KCV_P_) decreased. PDA resulted in increases in KCV_i_ and KCV_P_. A ductile-to-brittle transition occurred from −90 °C to −190 °C. Cryogenic Charpy impact testing of the heterostructured material revealed inflections on impact load–deflection curves. The phenomenon contributed to an increase in KCV_P_, providing a longer crack propagation path. The heterostructured material possessed an excellent σ_m_-KCV combination in the temperature range between −90 °C and +20 °C.

## 1. Introduction

High- or medium-entropy alloys (H/MEAs) have drawn increasing attention as promising materials for structural applications [1,2,3,4] due to their superior combination of mechanical properties [5,6,7,8]. For instance, H/MEAs based on 3d transition metals (3D-TM H/MEAs) with a face-centered cubic (FCC) lattice structure achieve good toughness at room and cryogenic temperatures [9,10]. Recent developments in non-equiatomic 3D-TM H/MEAs, especially ones further doped with interstitial elements, have been associated with the metals attaining an outstanding combination of strength and ductility [10,11,12]. The improved mechanical characteristics of such alloys [13,14,15] are usually attributed to the activation of a wide range of strengthening (solid solution strengthening, dislocation strengthening, and grain-boundary and dispersion strengthening) [16] and deformation (dislocation slip, strain-induced martensitic transformation, and mechanical twinning) mechanisms simultaneously [17].

The value of stacking fault energy (SFE) determines the active deformation mechanisms and mechanical behavior of FCC alloys [18,19,20]. For instance, the SFE value of the non-equiatomic 3D-TM MEA (80-X)Fe-XMn-10Co-10Cr (hereinafter, the compositions are given in at.% unless stated otherwise) decreases to 6 MJ/m^2^ when the manganese content is reduced to 30%, causing the development of a strain-induced γ→ε martensitic transformation [9]. The addition of 0.5% carbon to the resulting 49.5Fe-30Mn-10Co-10Cr-0.5C alloy is accompanied by an increase in the SFE value to 18 MJ/m^2^ [14], which provokes the manifestation of multiple deformation mechanisms, in particular, mechanical twinning and strain-induced γ→ε martensitic transformation, and therefore excellent strain hardening. So, the 49.5Fe-30Mn-10Co-10Cr-0.5C alloy can be considered one of the most promising non-equiatomic 3D-TM H/MEAs for engineering applications.

The microstructure and mechanical properties of non-equiatomic 3D-TM H/MEAs subjected to conventional thermomechanical processing have been studied earlier in detail [21,22,23,24,25]. It has been established that good strength and high ductility is achieved in the 49.5Fe-30Mn-10Co-10Cr-0.5C alloy via the formation of heterogeneous structures during cold rolling and post-deformation annealing [14,15]. Yet, increased mechanical properties can be obtained in metallic materials using other techniques. For instance, cold rotary swaging (CRS) has recently been studied as a technique for the production of workpieces with high strength properties, impact toughness, and corrosion resistance [26,27,28,29]. CRS is a process of severe plastic deformation in which a cylindrical workpiece undergoes a reduction in diameter via high-frequency pulse hammer stroking. The non-uniform distribution of external stresses and plastic strain over the rod diameter during CRS is predicted by finite element simulations [30,31].

It has been shown that CRS to a reduction of 80–90% and subsequent low-temperature annealing at 600 °C of the 49.5Fe-30Mn-10Co-10Cr-0.5C alloy produce a heterogeneous structure over the cross-section of the rod [31]. The heterogeneous structure determines the increased tensile properties of the material and allows it to overcome the strength–ductility trade-off [32,33]. In this case, a twinned FCC matrix and dislocation-free grains with a 1 μm diameter are observed in the center, whilst an ultrafine-grained FCC microstructure is formed at the edge of the cylindrical workpiece.

However, little attention has been paid to the effect of such a heterogeneous structure on the mechanical behavior of non-equiatomic 3D-TM H/MEAs during Charpy impact testing at room and cryogenic temperatures, because the main body of studies have been devoted to FCC alloys with a uniform microstructure. Moreover, the cryogenic mechanical properties of heterostructured H/MEAs need to be evaluated for better understanding of their potential applications. Apparently, grain refinement and carbide precipitation during aging reduce the cryogenic Charpy impact toughness of FCC alloys due to the suppression of mechanical twinning [34,35,36]. Whereas mechanical twins contribute to high cryogenic Charpy impact toughness, plates of ε-matrensite deteriorate the dynamic properties [37]. Moreover, a gradual decrease in ductility at low temperatures also causes a lack of impact toughness [38]. On the other hand, heterostructured materials can achieve increased toughness [39,40], which is ascribed to crack bridging, inflection, and/or delamination [41]. Thus, the aim of this work is to determine the effect of a heterogeneous structure obtained via CRS and post-deformation annealing on the dynamic mechanical properties and fracture mechanisms of a non-equiatomic 49.5Fe-30Mn-10Co-10Cr-0.5C 3D-TM MEA.

## 2. Materials and Methods

### 2.1. Initial and As-Processed Condition

**Figure 1:**
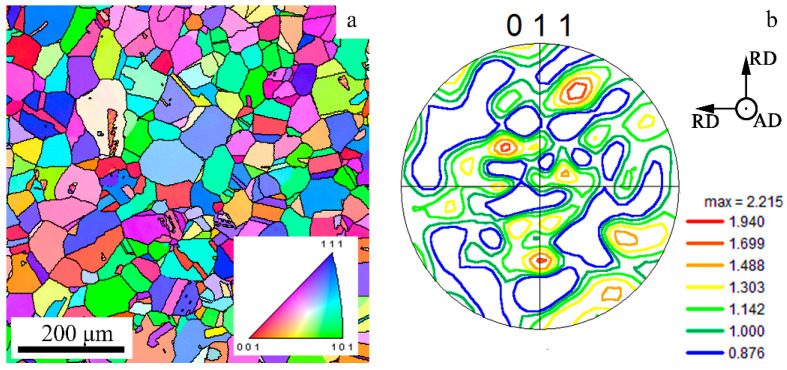
(**a**) IPF map and (**b**) pole figure of the 49.5Fe-30Mn-10Co-10Cr-0.5C alloy in the initial condition. RD—radius direction; AD—axial direction.

**Figure 2:**
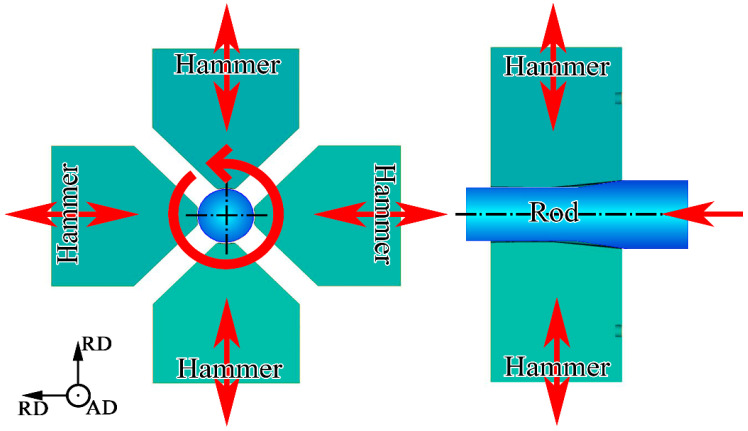
Scheme of CRS processing. RD—radius direction; AD—axial direction.

The chemical composition of the non-equiatomic 49.5Fe-30Mn-10Co-10Cr-0.5C (at.%) alloy is presented in Table 1. It was estimated using a Foundry Master OE750 spectrometer (Hitachi, Uedem, Germany). The ingot of the material under study was derived from pure components via vacuum induction melting. Then, hot forging was carried out in a temperature range of 950–1200 °C to attain a rod with a diameter of 42 mm. Equiaxed grains of the γ-phase with an average diameter of ~50 μm were observed in the obtained rod (Figure 1). The microstructure was uniform and weak-textured. The same microstructure was detected in the core, in the middle of the radius, and at the edge of the rod. Afterwards, the rod was subjected to CRS in accordance with the previously tested regimes [42,43,44]: a feed rate of 180 mm per minute, a strike frequency of 1000 beats per minute, and a workpiece rotation speed of 25 rotations per minute. Water cooling was performed during the CRS process. The scheme of CRS is shown in Figure 2. CRS was conducted with the following sequence: from ∅42 mm to ∅38 mm; from ∅38 mm to ∅32 mm; from ∅32 mm to ∅26 mm; from ∅26 mm to ∅18 mm; from ∅18 mm to ∅15.5 mm; and from ∅15.5 mm to ∅12 mm, which corresponded to a swaging reduction of 18% (CRS18), 42% (CRS42), 62% (CRS62), 82% (CRS82), 85% (CRS85), and 92% (CRS92), respectively. After CRS92, the material under study was annealed at 500 °C (ANN500), 600 °C (ANN600), 700 °C (ANN700), 800 °C (ANN800), and 900 °C (ANN900) for 10 min with subsequent air cooling.

### 2.2. Microstructure and Texture Characterization

The microstructure and texture of thin foils were characterized via transmission (TEM) and scanning electron (SEM) microscopy. TEM characterization was carried out using JEOL JEM-2100 (JEOL, Tokyo, Japan) at an accelerating voltage of 200 kV. Inverse pole figure (IPF) maps, phase maps, and direct pole figures were constructed using electron backscattered diffraction (EBSD) analysis data collected on a FEI Quanta 600 (FEI Company, Hillsboro, OR, USA) or Tescan Mira 3 (Tescan, Brno, Czech Republic) scanning electron microscope at an accelerating voltage of 20 kV. Analysis of X-ray diffraction (XRD) was conducted using a Rigaku Ultima-IV X-ray diffractometer (Rigaku, Tokyo, Japan) in CuKα radiation.

### 2.3. Charpy Impact Testing

**Figure 3:**
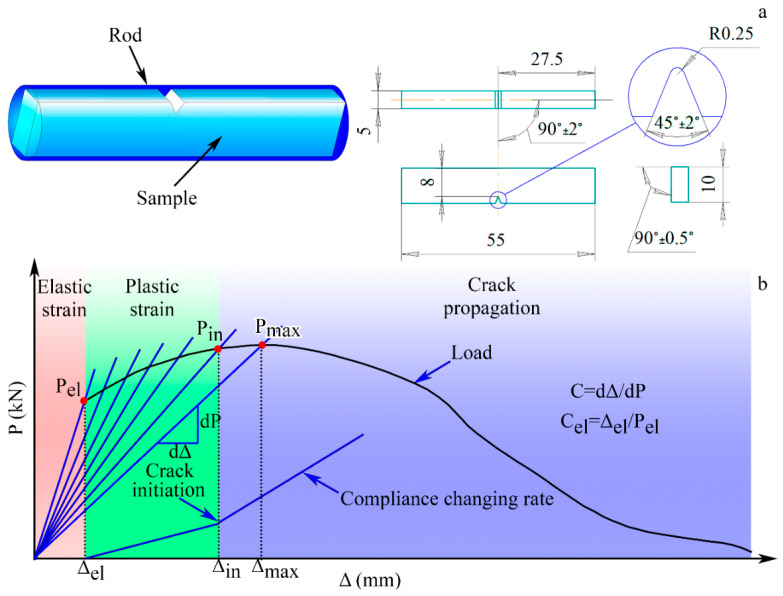
(**a**) Cutting scheme and dimensions of a Charpy V-notch specimen; (**b**) application of the CCR method.

Charpy V-notch specimens with dimensions of 5 × 10 × 55 mm^3^ were cut along the rod axis in accordance with the cutting scheme in Figure 3a. Charpy impact tests were performed on an Instron 450 J pendulum impact tester (Instron, Norwood, MA, USA) at room and cryogenic temperatures (−20 °C, −60 °C, −90 °C and −190 °C). Fracture surfaces were studied on a FEI Quanta 600 scanning electron microscope (FEI Company, Hillsboro, OR, USA) at an accelerating voltage of 30 kV. The maximum tensile stress ($\sigma_{m}$) perpendicular to the minimum cross section of a Charpy V-notch specimen was calculated using the following equation [45]:

(1) $$\sigma_{m}=\frac{\beta LP_{max}}{2C_{ƒ}{\left(W-a\right)}^{2}B}$$

where L—the distance between supports (=40 mm); $P_{max}$—the maximum load (kN); W—the width of the specimen (=10 mm); B—the thickness of the specimen (=5 mm); a—the notch depth (=2 mm); $C_{ƒ}$—the constant coefficient (=1.363 for an ASTM hammer); and β—the constant (β = 2 [46]).

To distinguish the contributions of crack initiation and crack propagation in the value of Charpy V-notch impact toughness, the compliance changing rate (CCR) method was applied [47]. According to the CCR method, the crack initiation point was found on the CCR plot (Figure 3b) that was calculated using the following equation [47,48]:

(2) $$\frac{\Delta C}{C}=\frac{\left(C-C_{el}\right)}{C_{el}}$$

where $\frac{\Delta C}{C}$—the compliance changing rate; C—the current compliance; and $C_{el}$—the elastic compliance.

## 3. Results

### 3.1. Microstructure and Texture After CRS and Post-Deformation Annealing

**Figure 4:**
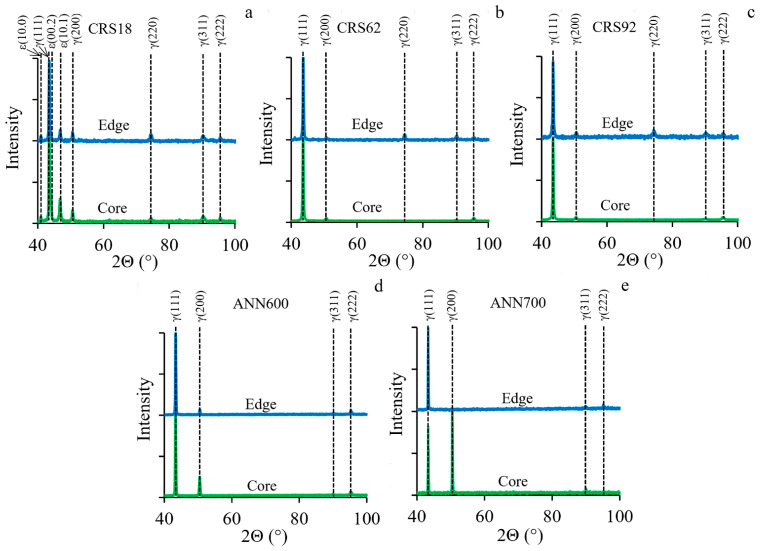
XRD patterns of the 49.5Fe-30Mn-10Co-10Cr-0.5C (at.%) alloy subjected to (**a**) CRS18, (**b**) CRS62, and (**c**) CRS92 and subsequent (**d**) ANN600 and (**e**) ANN700.

**Figure 5:**
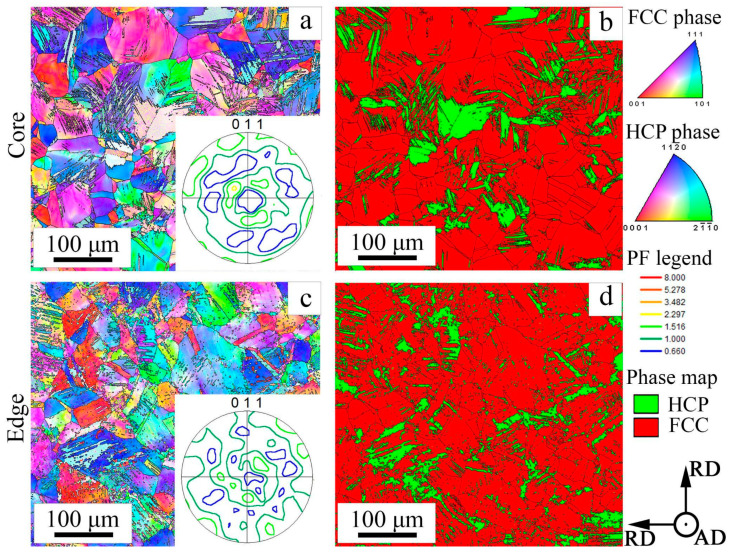
SEM-EBSD characterization of the 49.5Fe-30Mn-10Co-10Cr-0.5C alloy subjected to CRS18: (**a**,**c**) IPF maps and (**b**,**d**) phase maps. Pole figures are inserted in (**a**,**c**).

**Figure 6:**
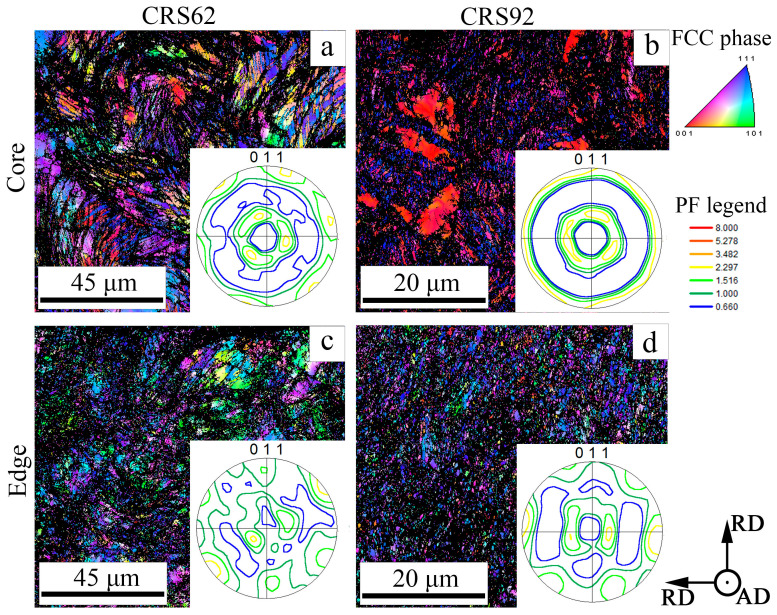
SEM-EBSD characterization of the 49.5Fe-30Mn-10Co-10Cr-0.5C alloy subjected to (**a**,**c**) CRS62 and (**b**,**d**) CRS92: IPF maps and pole figures (PFs).

**Figure 7:**
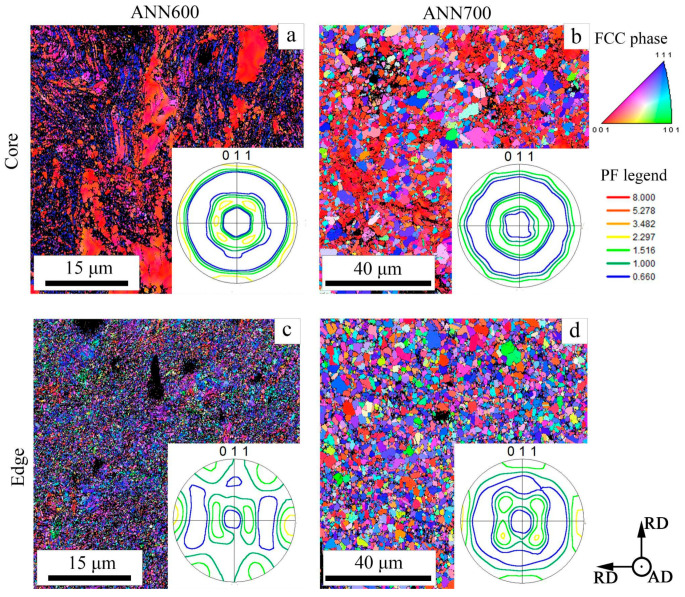
SEM-EBSD characterization of the 49.5Fe-30Mn-10Co-10Cr-0.5C alloy subjected to (**a**,**c**) ANN600 and (**b**,**d**) ANN700: IPF maps and pole figures (PFs).

**Figure 8:**
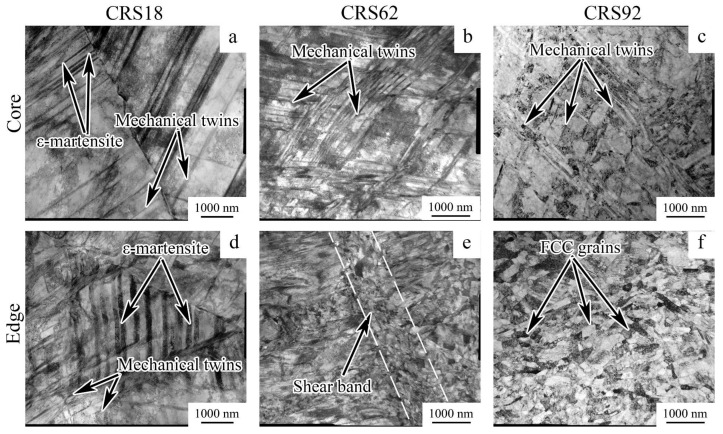
TEM characterization of the 49.5Fe-30Mn-10Co-10Cr-0.5C alloy subjected to (**a**,**d**) CRS18, (**b**,**e**) CRS62, and (**c**,**f**) CRS92.

The results of the XRD analysis, SEM-EBSD, and TEM characterization of the material after CRS and following annealing are shown in Figure 4, Figure 5, Figure 6, Figure 7 and Figure 8. Peaks of the γ-phase with the FCC structure and the ε-martinsite with the hexagonal close-packed (HCP) structure were recognized via XRD analysis after CRS18 (Figure 4a). CRS62-92 resulted in the single γ-phase condition (Figure 4b,c). After ANN600 and ANN700, only peaks of the γ-phase were detected again (Figure 4d,e). Meanwhile, a significant increase in the intensity of the (200)γ peak occurred in the core of the rod.

According to the results of EBSD analysis, CRS18 provoked the formation of the ε-martensite within the γ-matrix (Figure 5). However, in the rod core, the ε-martensite content was 18% (Figure 5b), whereas 9% of the ε-martensite was detected at the rod edge (Figure 5d). CRS62-92% was associated with a significant decrease in the ε-martensite content. Increasing CRS reduction resulted in the microstructure refinement, especially at the edge of the rod (Figure 6). ANN600 mostly preserved the as-swaged microstructure (Figure 7a,c). After ANN700, a globular fine-grained microstructure was attained over the cross section of the rod (Figure 7b,d).

During CRS, a pronounced duplex axial 〈111〉- and 〈100〉-texture of the γ-phase developed in the core of the rod (Figure 5a and Figure 6a,b). At the edge of the rod, the formation of a shear $B/\bar{B}$-texture of the γ-phase was observed (Figure 5c and Figure 6c,d). The same texture patterns were found after ANN600 (Figure 7a,c). After ANN700, the axial 〈111〉-texture of the γ-phase was slightly weakened (Figure 7b), while the axial 〈100〉-texture of the γ-phase was still strong. Furthermore, at the edge of the rod, the shear $B/\bar{B}$-texture of the γ-phase transformed into a Cube texture (Figure 7d).

TEM observations revealed that, in the core of the rod, CRS18 provoked mechanical twinning via a single system and the formation of parallel ε-martensite plates inside γ-grains (Figure 8a). Yet, at the edge of the rod, several twinning systems operated (Figure 8d). Plates of ε-martensite were also observed between packets of twins. CRS62 was accompanied by the development of multiple twinning in the core of the rod (Figure 8b), whereas a mostly lamellar microstructure formed at the edge (Figure 8e). Some shear bands can also be found herein. After CRS92, a twin-matrix microstructure was developed in the core of the rod due to the operation of several twinning systems (Figure 8c). An ultrafine-grained (UFG) microstructure was attained at the edge of the rod (Figure 8f). With a CRS reduction of up to 92%, the dislocation density increased from 1.3 × 10^11^ ± 1.7 × 10^10^ m^−2^ (in the initial condition) to 1.2 × 10^13^ ± 1.8 × 10^12^ m^−2^ in the rod core and 1.8 × 10^13^ ± 3.6 × 10^12^ m^−2^ at the rod edge. Meanwhile, in the CRS92 material, the size of the substructure elements was 654 ± 55 nm and 303 ± 10 nm in the core and at the edge of the rod, respectively.

**Figure 9:**
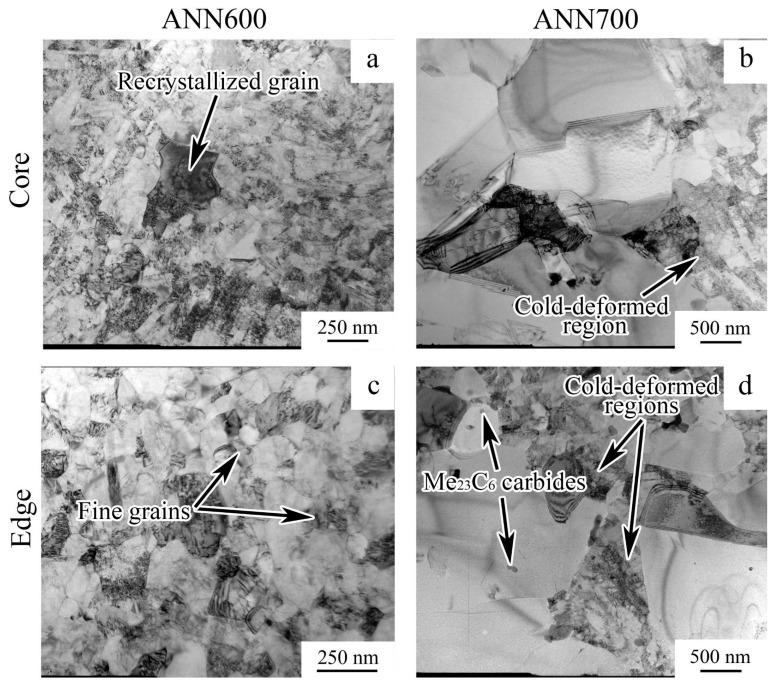
TEM characterization of the 49.5Fe-30Mn-10Co-10Cr-0.5C alloy subjected to (**a**,**c**) ANN600 and (**b**,**d**) ANN700.

Subsequent ANN600 caused static recovery and the onset of static recrystallization. So, the formation of fine grains with a low dislocation density (~200 nm) at the edge of the rod and larger recrystallized grains (~1 µm) in the core of the rod was observed (Figure 9a,b). In turn, ANN700 promoted static recrystallization over the cross-section of the rod (Figure 9c,d). Additionally, regions of the retained cold-deformed microstructure and Me_23_C_6_ carbides were found after ANN700 (Figure 9d).

### 3.2. Mechanical Behavior During Charpy Impact Testing

#### 3.2.1. Charpy Impact Testing at Room Temperature

**Figure 10:**
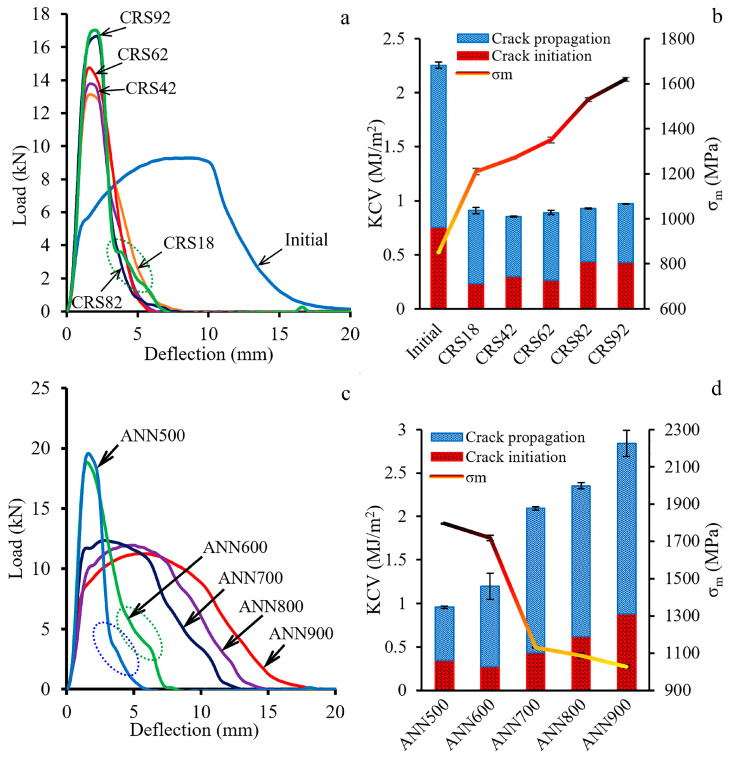
(**a**,**c**) Impact load–deflection curves and (**b**,**d**) dynamic mechanical properties (Charpy V-notch impact toughness (KCV) and maximum stress (σ_m_)) after CRS and post-deformation annealing. Dotted ellipses outline inflections in (**a**,**c**).

Impact load–deflection curves and dynamic mechanical properties (Charpy V-notch impact toughness (KCV) and maximum stress (σ_m_)) at room temperature after CRS and post-deformation annealing are presented in Figure 10. The impact load–deflection curve of the material in the initial condition showed pronounced strain hardening after yielding (Figure 10a). The region of the unstable crack growth [47,49] was smooth. CRS was accompanied by an increase in the maximum load value, while the strain-hardening stage deteriorated. Apparently, inflections were detected on the crack propagation stage during testing of CRS92 specimens. Multiple inflections during load descending were also found after ANN500 and ANN600 (Figure 10c). After ANN700, ANN800, and ANN900, the yield point again appeared on the impact load–deflection curves, followed by a strain-hardening stage.

A more than twofold decrease in KCV value was observed after CRS18 (Figure 10b). Although the KCV value was stable, the crack initiation energy consumption (KCV_i_) increased with CRS reduction, whilst the crack propagation energy consumption (KCV_p_) slightly decreased. With an increase in annealing temperature, KCV_i_ and KCV_p_ were enhanced simultaneously (Figure 10d). CRS drastically increased the σ_m_ value (Figure 10b). After post-deformation annealing, the σ_m_ value was reduced dramatically (Figure 10d).

#### 3.2.2. Charpy Impact Testing at Cryogenic Temperatures

**Figure 11:**
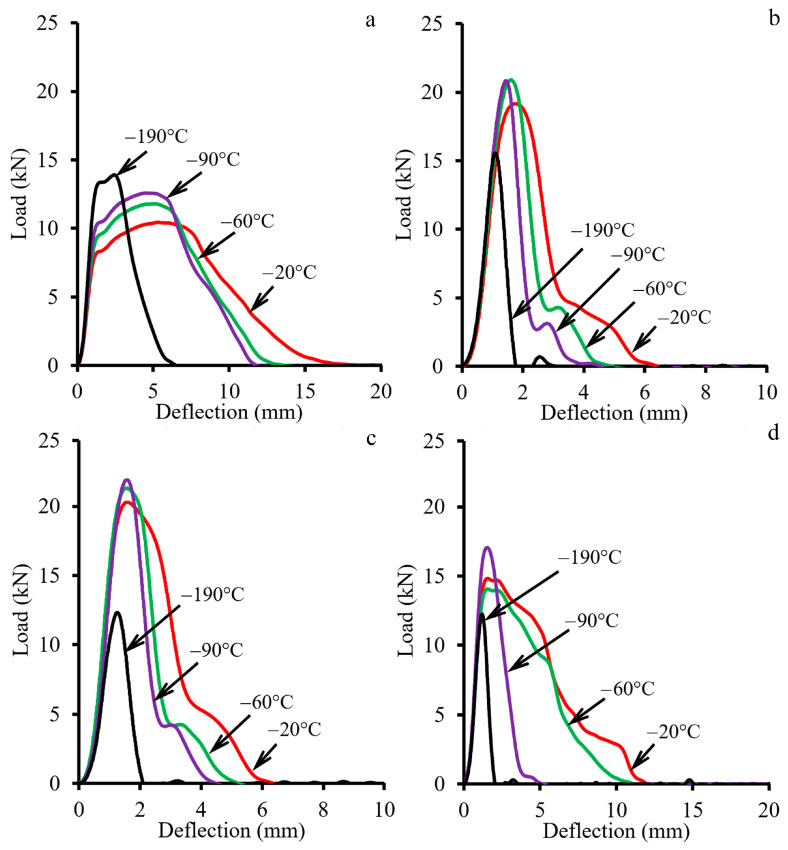
Impact load–deflection curves of the material under study in (**a**) the initial condition and after (**b**) CRS92, (**c**) ANN600, and (**d**) ANN700.

**Figure 12:**
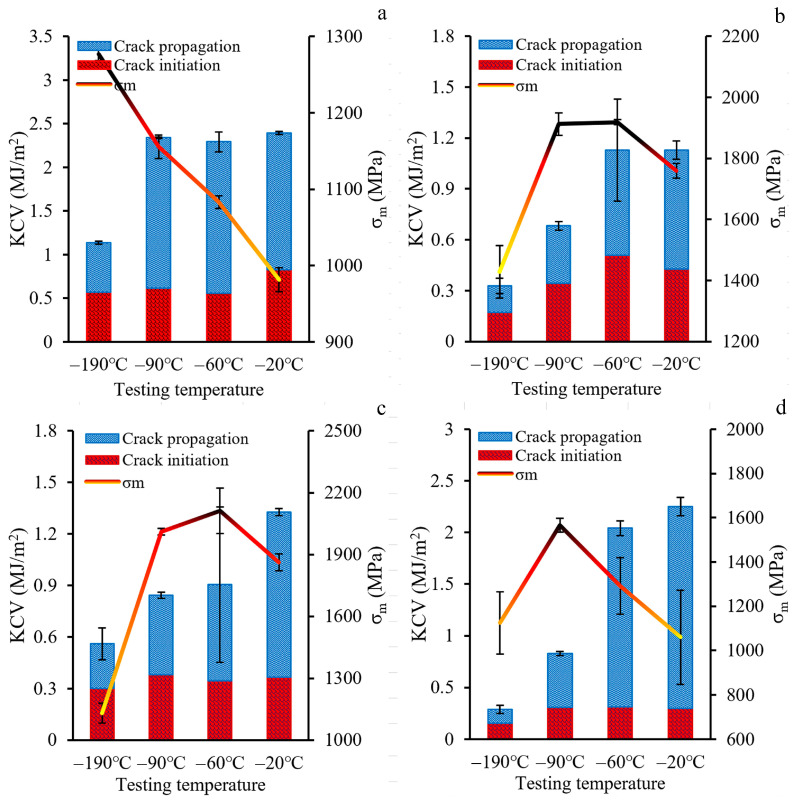
Charpy V-notch impact toughness (KCV) and maximum stress (σ_m_) of the material under study in (**a**) the initial condition and after (**b**) CRS92, (**c**) ANN600, and (**d**) ANN700 versus testing temperature.

The results of Charpy impact testing at cryogenic temperatures are shown in Figure 11 and Figure 12. For the initial condition, a decrease in testing temperature resulted in an increase in the maximum load value simultaneously with a “narrowing” of impact load–deflection curves (Figure 11a), causing a substantial increase in σ_m_ (Figure 12a). The KCV value was stable from −20 °C to −90 °C, yet, at −190 °C, KCV dramatically decreased due to the decrement of the crack propagation energy consumption. After CRS92 and ANN600, the material under study showed similar mechanical behavior, which was associated with its heterogeneous microstructure (Figure 10b,c). The additional peak during the crack propagation stage and a lack of strain-hardening capacity were found at a temperature range from −20 °C to −90 °C. At −190 °C, an obvious decrease in the maximum load and fast crack propagation were attained; this was accompanied by a drop in σ_m_ and KCV (Figure 12b,c). Therefore, a ductile-to-brittle transition occurred in a temperature range between −90 °C and −190 °C. However, ANN700 “stretched” the load–deflection curves (Figure 11d) and enhanced the KCV (Figure 12d), yet only in a temperature range from −20 °C to −60 °C. With a decrease in testing temperature to −90 °C and −190 °C, the impact load–deflection curves became “narrow” again and the mechanical properties decreased substantially. Hence, an embrittlement of the material subjected to ANN700 was observed at −90 °C and below.

**Figure 13:**
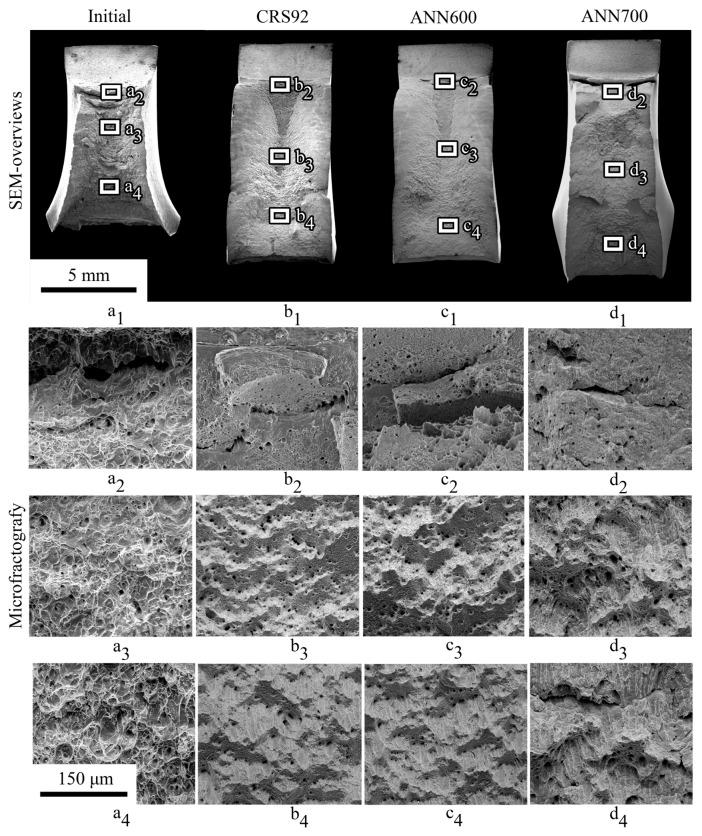
(**a_1_**–**d_1_**) SEM-overviews and (**a_2_**–**d_2_**,**a_3_**–**d_3_**,**a_4_**–**d_4_**) microfractography after Charpy impact testing at −20 °C.

**Figure 14:**
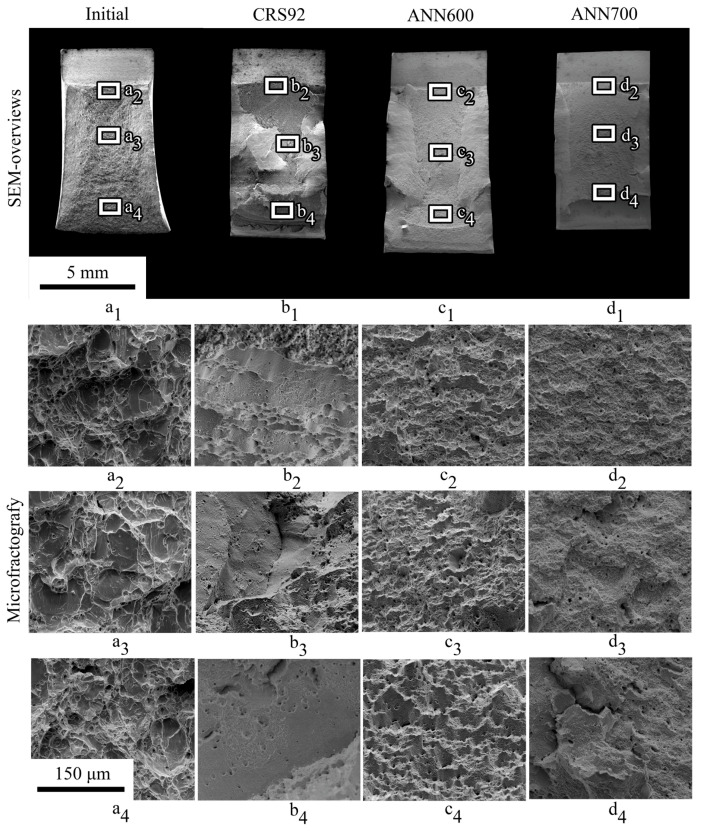
(**a_1_**–**d_1_**) SEM-overviews and (**a_2_**–**d_2_**,**a_3_**–**d_3_**,**a_4_**–**d_4_**) microfractography after Charpy impact testing at −190 °C.

Fracture surfaces of the Charpy V-notch impact specimens after tests at −20 °C and −190 °C are presented in Figure 13 and Figure 14, respectively. One can see that a decrease in testing temperature resulted in the suppression of the shear lip formation, thereby suggesting some decrease in the contribution of ductile deformation (Figure 13(a_1_–d_1_) and Figure 14(a_1_–d_1_)). However, in the initial condition, the material still had signs of a mainly ductile microfracture, both at −20 °C and −190 °C (Figure 13(a_1_–a_4_) and Figure 14(a_1_–a_4_)). After Charpy impact tests at −20 °C, secondary cracks were derived in the crack initiation zone (Figure 13(b_1_–d_1_)) of the as-processed material (after CRS92, ANN600, or ANN700). On the other hand, after Charpy impact tests at −190 °C, such secondary cracks were not found below the notch (Figure 14(b_1_–d_1_)). After CRS92 and ANN600, a mostly dimple morphology of the microfracture was observed in the rod core region at −20 °C (Figure 13(b_3_,c_3_)), whereas in the rod edge region, a stepwise faceted morphology of the surface could be seen (Figure 13(b_4_,c_4_)). Interestingly, after Charpy impact tests at −190 °C, the material subjected to CRS92 and ANN600 demonstrated a smoothed surface morphology, which was associated with a mostly brittle microfracture (Figure 13(b_3_,c_3_,b_4_,c_4_)). After ANN700, a rough stepwise microfracture was observed at −20 °C (Figure 13(d_3_,d_4_)), while a flattened microfracture could be seen at −190 °C (Figure 14(d_3_,d_4_)).

## 4. Discussion

The microstructure and texture evolution of the 49.5Fe-30Mn-10Co-10Cr-0.5C MEA during CRS and during further annealing was reported and discussed in detail in our previous work [31]. Here, we would like to focus on the interplay between the microstructure/texture and mechanical behavior of the alloy during Charpy impact tests. Although 3D M/HEAs are widely reported as materials with exceptional fracture and impact toughness [50,51,52,53], the links between their microstructure and impact resistance have not been well established, especially in heterostructured alloys.

In the program alloy, the observed evolution of the microstructure and texture during CRS and annealing affected the dynamic mechanical behavior and fracture mechanisms of the alloy during Charpy impact tests. CRS provoked a substantial increase in σ_m_ (Figure 10b), which was associated with a work-hardening effect due to microstructure refinement and an increase in dislocation density (Figure 5, Figure 6 and Figure 8). Interestingly, after ANN500 and ANN600, the σ_m_ value was higher than that after CRS92 (Figure 10b,d). Meanwhile, TEM and SEM-EBSD observations revealed that the microstructure and texture after ANN500 and ANN600 (Figure 8a,b) were mostly similar to those of the CRS92 material (Figure 6b,d), except for slight changes due to partial static recovery and the onset of static recrystallization. Apparently, the observed strengthening can be due to the effect of atomic segregations [54,55] resulting in dislocation pinning and hence the increased strength value (aging effect) [56]. With a further increase in annealing temperature, static recrystallization (Figure 9c,d) caused a significant decrease in the σ_m_ value (Figure 10d).

A decrease in KCV after CRS18 can be ascribed to a lack of strain hardening (Figure 10b) because of the microstructure fragmentation by mechanical twins and ε-martensite plates, as well as dislocation cells (Figure 8a,d). The microstructure refinement inhibited the impact energy dissipation and, therefore, decreased energy consumption during crack initiation and propagation [35]. Further CRS was associated with stabilization of the KCV value. However, the contribution of KCV_i_ increased due to strengthening. On the other hand, KCV_P_ decreased, which might be due to the suppression of plastic deformation and energy dissipation during crack growth. Further annealing resulted in increases in KCV_i_ and KCV_P_ via the enhancement of strain hardening and, thereby, energy dissipation.

The cutting scheme of Charpy V-notch specimens (Figure 3a) ensured that crack propagation occurred throughout the whole cross-section of the rod. Crack initiation was expected from the notch tip along the direction of the pendulum motion where the tensile stress mostly operated [57]. Certainly, the KCV_i_ value depended on the strengthening and strain-hardening of the material under loading, which were ascribed to its intrinsic toughening mechanisms [41,58]. Moreover, crack branching in the crack initiation zone resulted in additional energy consumption [59]. During subsequent crack growth, inflections on the obtained impact load–deflection curves after CRS92, ANN500 and ANN600, which resulted in an increase in the KCV_p_ value, can be observed (Figure 10a,c). The inflections can be attributed to the extrinsic toughening mechanisms, as well as crack bridging and delamination, which decreased local stress at the crack tip [41,58]. According to the results of SEM-EBSD analysis, the core of the rod possessed a pronounced axial 〈111〉-texture. The {111}<110> direction was preferable for crack propagation within the γ-phase [60]. Thus, in the rod core, crack growth occurred in the impact direction. On the other hand, at the edge of the rod, the γ-phase had a shear $B/\bar{B}$-texture (Figure 6d and Figure 7c). In this case, the failure direction corresponded to the shear plane [61], which was tangential to the rod surface [62].

To sum up, when the crack grew throughout the cross-section of the heterostructured rod, the crack inflections were derived from two regions with various texture patterns (the core and edge of the rod), which increased the KCV_p_ value via providing a longer crack propagation path and producing new fracture interfaces [41]. Yet the development of static recrystallization during ANN700 resulted in the weakening of the observed texture heterogeneity and thereby the demolishing of the inflection phenomenon (Figure 10c). Furthermore, the coarsening of the microstructure facilitated plastic deformation and increased energy consumption, i.e., the KCV_i_ and KCV_p_ values [35].

Charpy impact tests at cryogenic temperatures after CRS92 and ANN600 revealed pronounced inflections on impact load–deflection curves at a temperature range from −20 °C to −90 °C. Obviously, the inflections corresponded to a change in the fracture mechanism when the crack transitioned from the rod core region with an axial 〈111〉-texture of the γ-phase to the rod edge region with a shear $B/\bar{B}$-texture of the γ-phase. Therefore, in the rod core region, a dimple fracture was observed (Figure 13(b_3_,c_3_)) due to the nucleation and coalescence of microvoids. On the other hand, in the rod edge region, the stepwise morphology of the fracture surface was associated with an alternate crack growth along and across axis-oriented fibers (Figure 13(b_4_,c_4_)). Such changes in the crack direction provided a longer crack propagation path and produced new fracture interfaces (Figure 14(b_1_,c_1_)). At −190 °C, flat surfaces were evident on different length scales (Figure 14(b_1_–b_4_,c_1_–c_4_)). This phenomenon can be associated with embrittlement via a decrease in strain-hardening ability and hence energy dissipation during Charpy impact tests, which was also observed in high-Mn austenitic steels earlier [63].

**Figure 15:**
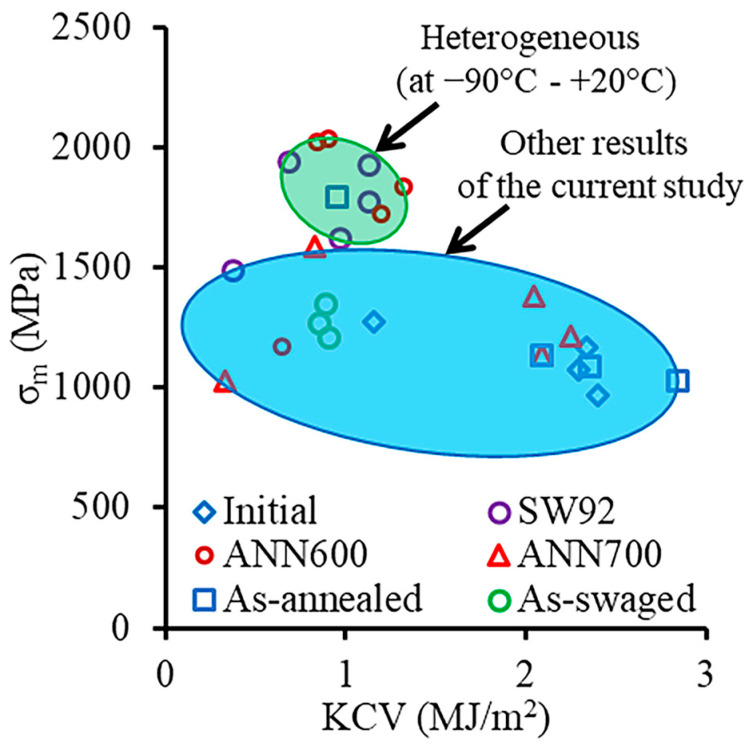
Maximum stress (σ_m_)–Charpy V-notch impact toughness (KCV) combination of the 49.5Fe-30Mn-10Co-10Cr-0.5C alloy.

Thus, the heterostructured material (after SW92, ANN500, and ANN600) achieved an excellent combination of σ_m_ and KCV at a temperature range between −90 °C and +20 °C (Figure 15). The increased KCV value was achieved via the effect of crack inflections between layers with various texture patterns, whereas the improved σ_m_ value was derived mostly from microstructure refinement and high dislocation density. The proposed thermomechanical treatment enabled us to produce large cylindrical parts for the automotive, nuclear, and airspace industries. Due to its excellent dynamic mechanical properties at low temperatures, parts made from the heterostructured material can be exploited in temperate, continental, and polar climate zones.

## 5. Conclusions

The effect of a heterogeneous structure obtained by cold rotary swaging (CRS) and post-deformation annealing on the dynamic mechanical properties and fracture mechanisms of a non-equiatomic 49.5Fe-30Mn-10Co-10Cr-0.5C (at.%) 3D-TM MEA at room and cryogenic temperatures was studied. The following conclusions were drawn:

1. CRS to a reduction of 92% developed a heterogeneous structure consisting of a twinned γ-matrix in the rod core and an ultrafine-grained microstructure of γ-phase at the rod edge. Subsequent annealing at 600 °C was accompanied by the static recovery and nucleation of γ-grains with low dislocation density. Higher annealing temperatures (700 °C and more) provoked static recrystallization. Duplex axial 〈111〉- and 〈100〉-textures of the γ-phase in the core of the rod and a shear $B/\bar{B}$-texture of the γ-phase at the edge of the rod were detected after CRS to a reduction of 92%. Annealing at 700 °C was accompanied by the transformation of the original axial 〈111〉-texture into a mostly axial 〈100〉-texture in the core of the rod. Meanwhile, the shear $B/\bar{B}$-texture of the γ-phase at the edge of the rod was transformed into a Cube texture.
2. CRS provoked a substantial increase in maximum stress (σ_m_) due to microstructure fragmentation by mechanical twins, ε-martensite plates, and dislocation cells. After annealing at 500 °C and 600 °C, σ_m_ was higher than that after CRS to a reduction of 92%, which was ascribed to the aging effect. With a further increase in annealing temperature, static recrystallization caused a significant decrease in σ_m_. A drop in Charpy V-notch impact toughness (KCV) after CRS to a reduction of 18% was ascribed to a lack of strain hardening. During further CRS, the KCV value was stable. However, the contribution of the crack initiation energy consumption (KCV_i_) increased, whilst the crack propagation energy consumption (KCV_P_) decreased. Subsequent annealing resulted in increases in KCV_i_ and KCV_P_ via the enhancement of the strain-hardening ability.
3. Charpy impact testing of the material subjected to CRS to a reduction of 92% at cryogenic temperatures and further annealing at 600 °C revealed pronounced inflections on impact load–deflection curves at a temperature range from −20 °C to −90 °C. The inflections corresponded to a change in the fracture mechanism when the crack transitioned from the rod core region with an axial 〈111〉-texture of the γ-phase to the rod edge region with a shear $B/\bar{B}$-texture of the γ-phase. In the rod core region, a dimpled morphology of the microfracture was observed. In contrast, in the rod edge region, a stepwise character of the microfracture was detected. A ductile-to-brittle transition was found from −90 °C to −190 °C. The heterostructured material possessed an enhanced σ_m_-KCV combination in the temperature range between −90 °C and +20 °C.

## Figures and Tables

**Table 1:** Chemical composition of the non-equiatomic 49.5Fe-30Mn-10Co-10Cr-0.5C alloy.

Component	Mn	Co	Cr	C	P	S	Si+Cu+Al+Ti	Fe
Content, at.%	29.51	10.91	9.52	0.47	0.001	0.002	<0.1	balance

## Data Availability

The original contributions presented in this study are included in the article. Further inquiries can be directed to the corresponding author.

## Abbreviations

The following abbreviations are used in this manuscript:

CRS	Cold rotary swaging
PDA	Post-deformation annealing
KCV	Charpy V-notch impact toughness
KCV_i_	Crack initiation energy consumption
KCV_P_	Crack propagation energy consumption
H/MEA	High- or medium-entropy alloy
3D-TM H/MEA	High- or medium-entropy alloy based on 3d-transition metals
HCP	Hexagonal close-packed
FCC	Face-centered cubic
TEM	Transmission electron microscopy
SEM	Scanning electron microscopy
IPF	Inverse pole figure
EBSD	Electron backscattered diffraction
XRD	X-ray diffraction
CCR	Compliance changing rate
